# Deep guided transformer dehazing network

**DOI:** 10.1038/s41598-023-41561-z

**Published:** 2023-09-15

**Authors:** Shengdong Zhang, Liping Zhao, Keli Hu, Sheng Feng, En Fan, Li Zhao

**Affiliations:** 1 Key Laboratory of Intelligent Informatics for Safety and Emergency of Zhejiang Province, Wenzhou University, Education Park Zone, Wenzhou City, 325035 Zhejiang Province People’s Republic of China; 2 Department of Computer Science and Engineering, Shaoxing University, Yuecheng District, Shaoxing City, 312000 Zhejiang Province People’s Republic of China

**Keywords:** Computer science, Information technology

## Abstract

Single image dehazing has received a lot of concern and achieved great success with the help of deep-learning models. Yet, the performance is limited by the local limitation of convolution. To address such a limitation, we design a novel deep learning dehazing model by combining the transformer and guided filter, which is called as Deep Guided Transformer Dehazing Network. Specially, we address the limitation of convolution via a transformer-based subnetwork, which can capture long dependency. Haze is dependent on the depth, which needs global information to compute the density of haze, and removes haze from the input images correctly. To restore the details of dehazed result, we proposed a CNN sub-network to capture the local information. To overcome the slow speed of the transformer-based subnetwork, we improve the dehazing speed via a guided filter. Extensive experimental results show consistent improvement over the state-of-the-art dehazing on natural haze and simulated haze images.

## Introduction

Image dehazing is a hot topic in classic computer vision, whose goal is to restore a clean image from the input. The quality of the captured image is affected by the air particle, which absorbs the ray emitted from objects and reflects other light into the camera. We can describe the hazing process as:

1 $$\begin{aligned} I\left( x\right) =t\left( x\right) J\left( x\right) +\left( 1-t\left( x\right) \right) A, \end{aligned}$$

where *I* is the input hazy image, and *J* is the corresponding clean image, *t* represents how much the light reflected from objects is received by the camera, *A* is the air-light.

In the traditional image dehazing, computing the transmission map and air-light is a highly ill-posed problem if there is no extra information available. To address dehazing problem, a lot of dehazing methods are designed based on the various types of priors^1–6^ or additional information^7–9^. Requiring additional information restricts the application scope of these methods. The priors used for dehazing maybe fail in some cases, such as images containing white objects or the sky. To boost the robustness of dehazing methods, deep learning-based methods^10,11^ are introduced to predict the transmission map. But the dehazing performance of these methods is influenced by the precision of the estimated transmission map. To overcome this problem, some End-to-End deep learning dehazing methods^12–20^ are proposed. Li et al. fuse the transmission map and airlight into a new parameter and design a low-time consumption dehazing method. Qu et al. designed a dehazing method, which transfers the dehazing problem into a transferring problem. Liu et al.employ the attention mechanism and multi-scale network to boost dehazing performance. Dong et al. employ boosting strategy and dense features fusion^21^ to design a dehazing network. Zhang et al. propose a transmission map guided dehazing network^22^. Song et al. propose a wavelet-based dehazing method^23^. Although these methods have their great power in dehazing, we note the performance can be further boosted by introducing a model which can capture long dependency.

CNN has shown its effectiveness in low-level computer vision tasks, while transformers have shown great power ability for high-level computer vision tasks. Recently some works although introduce it into low-level computer vision tasks^24^. The prior work^25^ introduced a transformer into a computer vision task and achieved an impressive performance, which shows the potential of computer vision tasks. However, the computational burden of the transformer is very high, which limits its application of transformer. To boost the dehazing performance, Dehamer^26^ propose a transformer-based module to estimate the density of haze, and then combine it with CNN features to obtain the final dehazed results. However, Dehamer ignores the information in hazy images, and cannot achieve a high dehazing for dense hazy images. Furthermore, Dehamer inherits the problem of the transformer, which has a high time complexity. Zhao et al. propose a Pyramid dehazing network^27^, which can extract large contextual information. However, this work also inherits the limitation of CNN. The proposed model extracts the large contextual information using a transformer and reduces the consumption time using the deep guided filter.

To address this issue, we propose a novel highly efficient dehazing method based on the transformer and guided filter, which is called Deep Guided Transformer Dehazing Network (DGTDN). Haze is depend on the distance between the camera and the objects, which results in the haze density being different from pixel to pixel. The distribution of haze is global, which is hard for CNN to capture the long distance. To capture the long dependency, we design a transformer-based model to capture the global information of haze. However, the transformer cannot capture the local information well. To deal with this case, we propose a lightweight CNN sub-network to capture the local information. Based on the advance of the transformer and CNN, we propose to restore the global haze-free image with the transformer and then refine the details with the CNN sub-network. To achieve the goal of further improving the dehazing speed, we introduce the guided filter to reduce the dehazing time. The contributions of the DGTDN can be summered as follows:

1. We introduce transformer-based sub-network to restore the coarse haze-free image and then the details are refined via a CNN-based sub-network. Restoring the coarse haze-free image depends on global information while refining the details needs more local information, which encourages us to design such a dehazing model using CNN and a transformer.
2. We introduce the guided filter to improve the dehazing speed. Transformer is time-consuming, which may limit the application of transformer-based dehazing methods. We address this issue by introducing the guided filter into the proposed model. We reduce the input size of the transformer, which reduces the execution time of the transformer-based model.
3. We do extensive experiments to show the superiority of the proposed method on natural hazy images and simulated hazy images. We also conduct ablation studies to show the effectiveness of the proposed modules.

## Related work

We show some previous works related to dehazing. In this paper, we divide the related dehazing works into two groups, which include learning-based and prior-based methods.

### Learning-based dehazing methods

The CNN-based methods have swept the computer vision tasks^28–30^. With the development of CNNs, a lot of works^10–15,17,31–36^ attempt to solve dehazing using deep learning models. These dehazing methods often attempt to compute the key factor of the physical model or the corresponding haze-free image directly. The works^10,11^ employ CNN model to compute the transmission map. However, these methods may boost the error of the transmission map and result in poor dehazing results. To deal this problem, End-to-End dehazing methods^12–15,17,31–34,37–40^ are proposed. For example, Zhang et al. design a CNN model that incorporates the physical model. Li et al. propose an all-in-one dehazing model^13^, which fuses the transmission map and airlight into a new parameter. Liu et al. design a novel dehazing model^20^ based on attention and multi-scale network. However, all these dehazing methods are based on CNNs, which are limited by the local property of convolution. To capture the long dependency of hazy images, Guo et al. propose a transformer-based dehazing method^26^, which employs the transformer-based encoder to capture the density of haze. Different from the above-mentioned methods, we overcome the problem of CNN by introducing the transformer block into the dehazing model, which can capture the long-range dependency. Some works note the difference between the simulated hazy and real hazy images, which results in a drop of dehazing performance on real hazy images when the model is trained with simulated hazy images. To address these issues, PSD^17^ proposes to combine the traditional priors to improve the dehazing quality of real hazy images. Domain adaptation dehazing method (DA)^19^ improves the dehazing quality on real hazy images by converting simulated hazy images into real hazy images. We note that these methods are hard to train. Furthermore, the proposed method focuses on improving the learning ability on simulated hazy images, which has a different goal from PSD and DA.

### Prior-based dehazing methods

To address the ill-posed of single image dehazing, a lot of prior-based dehazing methods^1–6^ or additional information^7–9^ has been proposed. These methods discover the prior based on the statistical analysis of clean images or hazy ones. The famous work is Dark Channel Prior (DCP), which is derived from the observation that a clean image patch contains at least one pixel that has a channel value close to zero. Zhu et al. discover a color attenuation prior^5^, which is that the divergence between intensity and saturation positively is correlated to the depth. Fattal et al.^2^ use a color-line prior to removing haze. Berman et al. find a haze-line prior^4^ based on the observation that one haze-free image can be presented by a small number of color clusters. However, all these priors are simple, and cannot be held in real word complex scenes.

### Transformer for vision tasks

Natural language processing (NLP) has applied Transformer^41^ to capture long dependency and improved the performance of learned models. Transformer shows its effectiveness in NLP and image classification task^25^ also employs Transformer to improve the performance. With the success of Vision Transformer (ViT)^25^ and its follow-ups^42,43^, researchers have shown the potential of transformers to image segmentation^43^ and object detection^42^. Although visual transformers have shown their success in visual tasks, it is hard to directly apply it in single image dehazing. First, Transformers often depend on large-scale datasets. However, there is no existing large-scale dataset to train a transformer-based for image dehazing. Second, it is hard to capture local representation for transformers, which may result in the loss of image details. To overcome this issue, we proposed combining the advantage of CNN and transformer to capture the local texture and global structure jointly to boost the dehazing quality.

**Figure 1:**
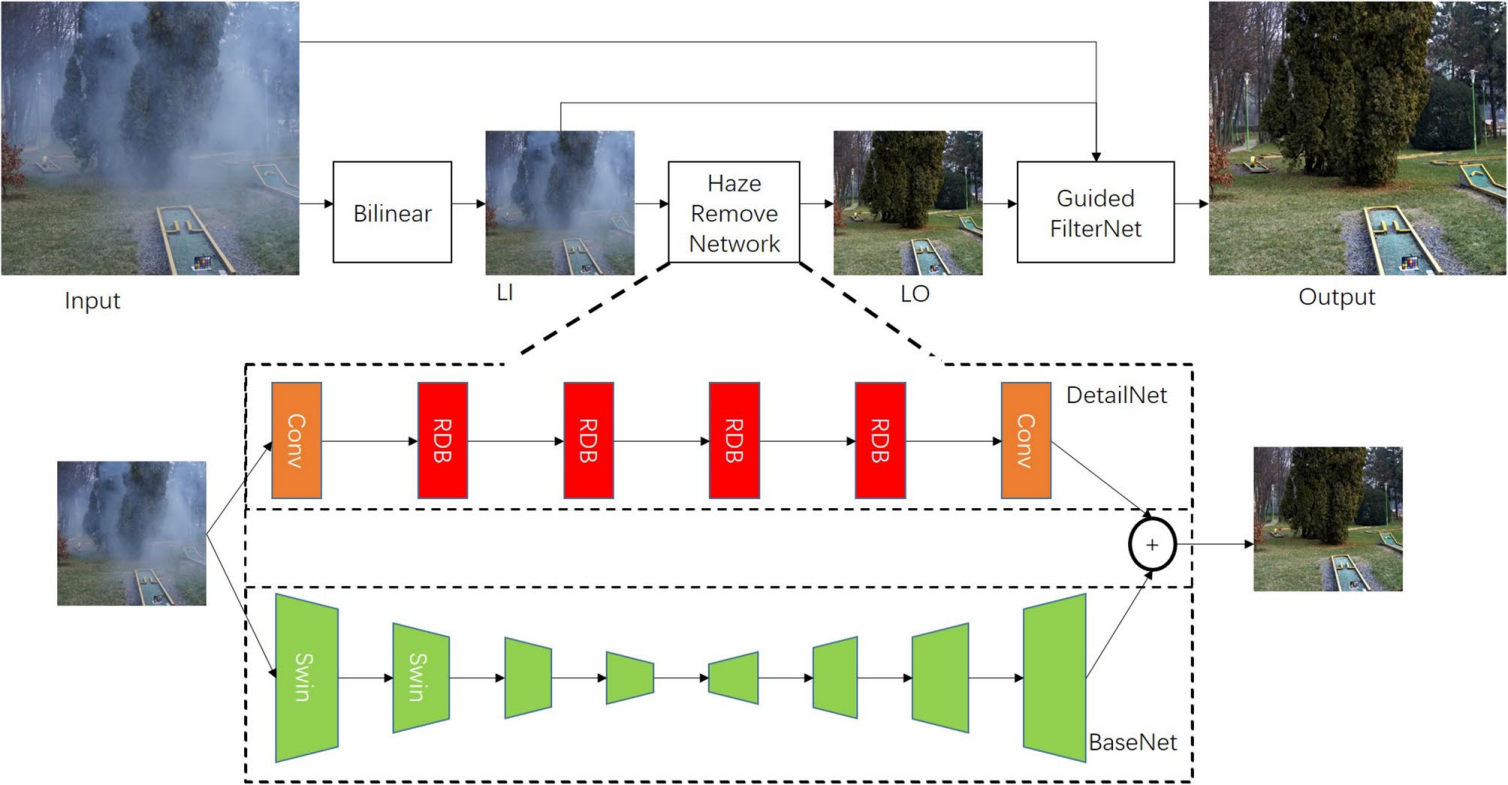
The rough structure of the Deep Guided Transformer Dehazing Network. The proposed network contains main three parts: BaseNet, DetailNet, and GuidedFilterNet. Swin represents the Swin-Block, which is used to enlarge the receptive field of the proposed model. Bilinear represents the bilinear downsampling. LI is the output of the bilinear downsampling. LO represents the output of the haze remove network, which is a low-resolution dehazing result.

## Methodology

In this section, we explain the motivation behind Deep Guided Transformer Dehazing Network (DGTDN) and then show the details of DGTDN. The structure of the proposed DGTDN is shown in Fig. 1, which consists of three parts. The first part is BaseNet, which is used to estimate the baselayer of the low-resolution dehazed result. The second part is DetailNet, which is used to estimate the missed details of base layer. The low-resolution dehazed result is generated by adding the base layer to the detail layer. The third part is GuidedFilerNet, which obtains the final high-quality dehazed result by upsamping the low-resolution dehazed result.

### Motivation

The thickness of the haze is dependent on the depth of the objects, which results in the distribution of haze is global information. Based on the fact that the dehazing task needs to restore the image details, which is dependent on the local features. Single image dehazing is dependent on global and local features^44^. The transformer has shown its ability to capture long-range dependency, which is critical to improve the dehazing quality. However, the transformer cannot capture the local feature details which leads to coarse details for dehazing. According to the prior works^45^, CNN can provide local connections and capture local features. It is known to all that transformer-based methods are time-consuming. To reduce the inference time, we propose to introduce the deep guided filter into the dehazing network. Based on the above analysis, we combine the advantages of CNN, transformer, and deep guided filter to boost the dehazing quality and reduce the running time. In this paper, we propose a Deep Guided Transformer Dehazing Network (DGTDN). DGTDN consists of BaseNet, DetailNet, and GuidedFilerNet. BaseNet is designed to capture long-rang dependency and restore the coarse haze-free image. DetailNet is designed to capture the local features and restore the image details. GuidedFilterNet is designed to enlarge the low-resolution dehazed result and reduce the dehazing time.

### The structure of the proposed model

Based on the motivation in subsection 3.1, we introduce the CNN, transformer, and guided filter into the proposed dehazing network. As shown in Fig. 1, we propose a model containing three parts: BaseNet, DetailNet, and GuidedFilerNet. We enlarge the details of haze remove network, which consists of BaseNet and DetailNet. As shown, the proposed model process a hazy image and outputs a high-resolution dehazed result via series steps: (1) Downsampling the input hazy image via bilinear downsampling, and obtaining a low-resolution haze image, we mark it as LI; (2) Feeding the LI into haze remove network, and obtaining a low-resolution dehazed result, we mark it as LO; (3) Feeding the LI, input hazy image, and LO into the GuidedFilterNet, and obtain the final high-resolution deazed result. Next, we introduce the BaseNet, DetailNet, and GuidedFilterNet in detail.

#### BaseNet

The BaseNet consists of an encoder that extracts features and a decoder that restores the haze-free image. The encoder contains four stages, and the decoder also contains four stages. Specifically, each encoder stage contains one transformer block, which followed one down-sampling layer. Similar to the encoder stage, each decoder stage contains one transformer block, which is followed by one up-sampling layer. The down-sampling layer is designed to downscale the size of feature maps, which is implemented by $3 \times 3$ convolution with stride 2. The up-sampling layer is designed to enlarge the size of feature maps, which is implemented by $2 \times 2$ transposed convolution operation with stride 2. The input of the BaseNet is a low-resolution version of a hazy image. The low-resolution hazy image is generated by using a bilinear, which is used to obtain a hazy image with half the size of the original input. We define the output of BaseNet as follows:

2 $$\begin{aligned} \hat{B} =BaseNet(I_l), \end{aligned}$$

where *BaseNet* is the BaseNet, the $I_l$ is the low-resolution of input hazy image, $\hat{B}$ is the base layer of a dehazed result.

#### DetailNet

**Figure 2:**
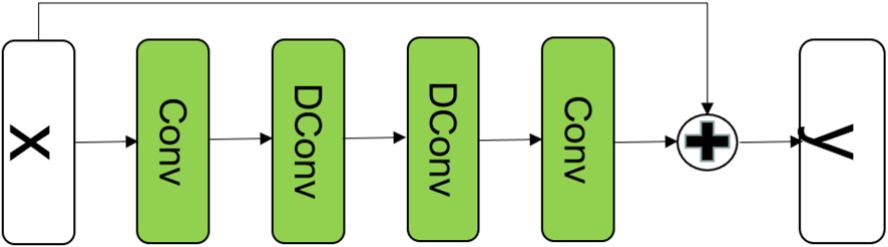
The structure of the Residual Dilation Block (RDB).

The DetailNet is designed to restore missed details. The DetailNet contains four Residual Dilation Blocks (RDBs), whose structure is shown in Fig. 2. Each RDB contains two common convolution layers and two dilation convolution layers. We pass the low-resolution input hazy into the DetailNet and obtain the detail layer.

3 $$\begin{aligned} \hat{D} =DetailNet(I_l), \end{aligned}$$

where *DetailNet* is the DetailNet, $\hat{D}$ is the detail layer of a dehazed result.

After obtaining the structure layer of dehazed result and the image detail layer, we can obtain the dehazed result as follow:

4 $$\begin{aligned} \hat{H}_l =\hat{B}+\hat{D}, \end{aligned}$$

where $\hat{H}_l$ represents the predicted low-resolution haze-free image.

#### GuidedFilterNet

GuidedFilterNet is based on the guided filter, which is based on the local linear model. We can express the local linear model as:

5 $$\begin{aligned} q_o(l) =A_{\omega }I_g(l)+B_{\omega }, \forall l \in \omega , \end{aligned}$$

where $q_o$ is the output, $I_g$ is the guidance image, *l* is the location in $I_g$, $\omega$ is a local window in $I_g$ with radius *r*, $(A_{\omega }, B_{\omega })$ are the linear const coefficients in a local window. This model can preserve the edges in $q_o$ if $I_g$ has the edges, because that $\nabla q_o= \nabla I_g$. To obtain the $(A_{\omega }$ and $B_{\omega })$, we solve the problem (5) that reduces the difference between the output $q_o$ and the filtering input *p*. To solve the problem (5), we minimizes the error:

6 $$\begin{aligned} E(A_{\omega }, B_{\omega })= \sum _{l \in \omega }((A_{\omega }I_g(l)+B_{\omega }- p(l))^2+\epsilon A_{\omega }^2), \end{aligned}$$

where $\epsilon$ is used to penalize large $A_{\omega }$, p is the filtering input.

We employ guided filter to perform joint upsampling, which receives a low-resolution hazy image, the corresponding low-resolution dehazed result, and the original hazy image as input, obtaining the final high-resolution dehazed result. Based on the local linear model, the relation between a low-resolution hazy image and the corresponding low-resolution haze-free image can be expressed:

7 $$\begin{aligned} H_l(i) =A_{\omega }^l I_l(i)+B_{\omega }^l, \end{aligned}$$

where $H_l$ is the low-resolution dehazed result and $I_l$ is the low-resolution hazy image, *i* is the index of the $I_l$. To obtain $A_{\omega }^l$ and $B_{\omega }^l$, we reduce the error between $\hat{H}_l$ and the $H_l$:

8 $$\begin{aligned} E(A_{\omega }^l, B_{\omega }^l )= \sum _{l \in \omega }((A_{\omega }^l I_l+B_{\omega }^l - \hat{H}_l)^2+\epsilon (A_{\omega }^l )^2), \end{aligned}$$

After obtaining $A_{\omega }^l$ and $B_{\omega }^l$, we simple the Eq. (7) to :

9 $$\begin{aligned} H_l =A^l.*I_l+B^l, \end{aligned}$$

where $.*$ is element-wise multiplication. Based on the local linear model, we also can express the relation between a high-resolution hazy image and the corresponding haze-free image as:

10 $$\begin{aligned} H_h =A^h.*I+B^h. \end{aligned}$$

Based on Eq. (10) and  (9), we can construct the relation between the high-resolution and the low-resolution hazy images. According to^46^, we can obtain the high-resolution $A^h$ and $B^h$ via bilinearly upsample:

11 $$\begin{aligned} A^h= & {} U(A^l) \end{aligned}$$

12 $$\begin{aligned} B^h= & {} U(B^l) \end{aligned}$$

Algorithm 1 lists the main steps of the guided filter in DGTDN. *U* is a bilinearly upsample operation, *Box* represents the box filtering. As shown in Fig. 1, GuidedFilterNet receives the output of haze remove network as input and enlarges the low-resolution dehazed result according to the original hazy image. In the proposed model, GuidedFilterNet interacts with haze remove network and bilinear downsampling, and performs a joint upsampling function. GuidedFilterNet is designed to enlarge the dehazed result and reduce the dehazing time of the proposed model.

**Algorithm 1:**
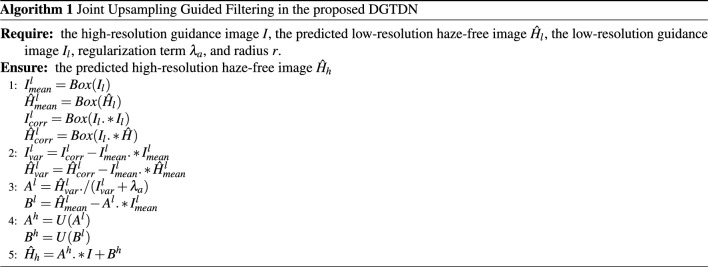
Joint Upsampling Guided Filtering in the proposed DGTDN.

### Loss functions

Loss functions are critical to obtaining high quality dehazing results. The proposed method can obtain two-scale dehazed results. To utilize this useful information, we propose a multi-scale content loss function:

13 $$\begin{aligned} \mathscr {L}_{\text {con}} =\frac{1}{N}\sum _{i=1}^{N}\left\Vert \hat{H}_h^i - H^{i}\right\Vert _1+\frac{1}{N}\sum _{i=1}^{N}\left\Vert \hat{H}_l^i - H_l^{i}\right\Vert _1, \end{aligned}$$

where *N* denotes the number of training samples, $\Vert \cdot \Vert _1$ denotes $L_1$ norm, $H_h$ is the ground-truth haze-free image, and $H_l$ is the low-resolution ground-truth. To make the predicted base layer similar to the low-resolution ground truth, we employ a $L_1$ loss between the low-resolution ground truth and the predicted base layer:

14 $$\begin{aligned} \mathscr {L}_{\text {baseloss}} =\frac{1}{N}\sum _{i=1}^{N}\left\Vert \hat{B}^i - H_l^{i}\right\Vert _1, \end{aligned}$$

where $\mathscr {L}_{\text {baseloss}}$ is defined as a base loss. To further boost the quality of dehazed result, we introduce perceptual loss to train the proposed model:

15 $$\begin{aligned} \mathscr {L}_{\text {perc}} =\frac{1}{N}\frac{1}{J}\sum _{i=1}^{N}\sum _{j=1}^{J}\left\Vert VGG(\hat{H}_h^i) - VGG(H_l^{i})\right\Vert _1, \end{aligned}$$

where *VGG* represents the VGG-16 model, which is a classic model trained on ImageNet, and *j* indicates which layer is used to estimate the perceptual loss.

Finally, we combine the perceptual loss, the multi- scale content loss, the base loss, and the perceptual loss to train the whole network, which can be defined as:

16 $$\begin{aligned} \mathscr {L}_o =\mathscr {L}_{\text {con}} +\lambda _1 \mathscr {L}_{\text {baseloss}}+\lambda _2 \mathscr {L}_{\text {perc}}, \end{aligned}$$

where $\lambda _1$ is used to determine the contribution of the base loss, and $\lambda _2$ is used to determine the contribution of the perceptual loss.

## Experimental results

In this section, we focus on showing the high performance of the proposed method. First, we introduce the implementation details of the proposed method and dataset. Second, we compare the proposed method with other dehazing methods on simulated haze images and real haze images. Third, we show the effectiveness of the proposed modules and loss functions.

**Table 1:** Details of the RDB.

layers	Conv1	Dconv2	Dconv3	Conv4
Size	3	3	3	3
Channels	16	16	16	16
Dilation rates	1	2	2	1

**Table 2:** Evaluation results of dehazed results using average PSNR/SSIM on the SOTS dataset from RESIDE^47^.

	BCCR	MSCNN	DehazeNet	CAP	DCP	NLD	AOD-Net	GFN	DCPDN	MSDFF	Dehamer	DGTDN
PSNR	16.88	17.57	21.14	19.05	16.62	17.29	19.06	22.30	15.86	33.75	36.36	36.68
SSIM	0.79	0.81	0.85	0.84	0.82	0.75	0.85	0.88	0.82	0.98	0.98	0.99

### Implementation details

In this subsection, we show the details of the proposed model. The proposed BaseNet is implemented based on the Swin-Transformer block. The configurations of the proposed RDB are listed in Table 1. The proposed DGTDN is implemented in a popular deep learning tool (PyTorch) using a single GPU ( TITAN V ) with 12GB memory. When training, we crop the training dataset into image patches with size $240\times 240$. The learning rate is set to 0.001 and then is decreased by 0.8 every 10000 steps. We set the batch size to 16. We employ the adam to train the proposed model and initialize the $\beta _1$ and $\beta _2$ to 0.5 and 0.999, respectively. We set $\lambda _1$ and $\lambda _2$ to 1.0 and 0.01, respectively.

According to the strategy adopted by^20,21,36^, ITS from RESIDE is chosen to train the proposed model and indoor hazy images from SOTS subset are used to evaluate the dehazing performance. In addition, we evaluate the performance on NH-HAZE.

### Experimental results on simulated hazy images

**Figure 3:**
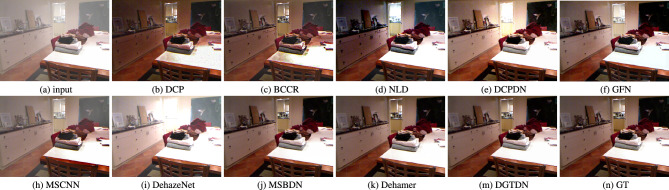
Visual results of some recently dehazing methods and the proposed method. The dehazed result obtained by other dehazing methods often retain haze or color distortion. The proposed method can remove haze more completely and obtain a more natural dehazing result.

In this part, we show the dehazing performance of the proposed DGTDN and other dehazing methods on the simulated indoor hazy images. Due to the fact, it is hard to find a ground truth haze-free image for a real haze image, simulated indoor hazy images are used to evaluate the dehazing performance. We show quantitative and visual dehazing results in Table 2 and Fig. 3. As shown in Table 2, traditional dehazing methods can obtain low quantitative results. Traditional dehazing methods derive prior from haze-free images, which may not be held by some hazy images. This is the main reason why traditional dehazing cannot achieve a high dehazing performance. The learning based dehazing methods include two kinds. The first is learning to predict transmission map, such as MSCNN^10^ and DehazeNet^11^. The second kind is learning to predict clean images directly, such as DCPDN^15^, GFN^12^, MSBDN^21^, and Dehamer^26^. The learning-based methods^10,11^ that learn the relationship between transmission map and hazy images. However, the relationship between transmission maps and dehazing quality is not highly correlated, which results in a low dehazing performance. End-to-end dehazing methods^12,15,21,26^ construct the relationship between hazy images and dehazed results. However, the dehazing ability of these models depends on the model capacity. The transformer-based dehazing method has a high model capacity and achieves the second dehazing performance. To summarize, the proposed method achieves outstanding performance among famous dehazing methods. As shown in Fig. 3, we note that traditional dehazing methods, such as DCP, NLD, and BCCR often have the problem of color distortion. The learning-based methods^10–12,15^ have the problem of retaining haze. Other leaning-based methods^21,26^ can obtain dehazed results that are similar to ground truth. The proposed can obtain high-quality visual dehazing results, which are more similar to ground truth.

**Table 3:** Evaluation results of dehazed results using average PSNR/SSIM on the dataset NH-HAZE^48^.

	DCP	BCCR	MSCNN	DehazeNet	NLD	AOD-Net	GFN	DCPDN	MSDFF	Dehamer	DGTDN
PSNR	12.35	12.15	17.72	11.76	12.01	17.42	15.17	15.86	16.21	19.25	19.86
SSIM	0.40	0.38	0.67	0.40	0.38	0.57	0.52	0.61	0.58	0.62	0.66

**Figure 4:**
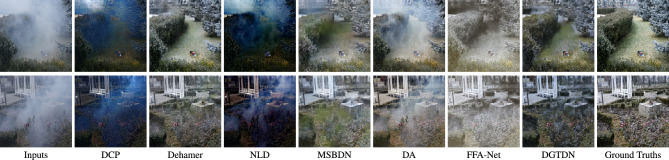
Visual results of dehazing methods on the dense non-homogeneous haze images^48^. The proposed method restores more haze-free images with clearer structures and textures.

**Figure 5:**
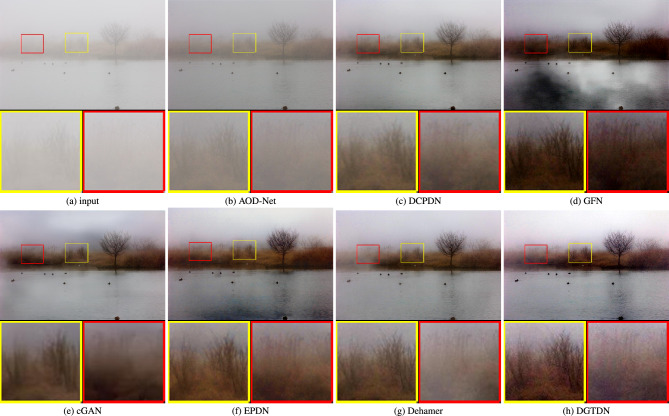
Visual results of some recently dehazing methods and the proposed method on lake scene with dense haze. The dehazed result obtained by other learning-based dehazing methods often retains haze. The proposed method can remove haze more completely and obtain a more natural dehazing result.

We also test the dehazing performance on NH-HAZE^48^, which is a widely used dataset. NH-HAZE is a famous dehazing dataset, which contains non-homogeneous haze. The non-homogeneous haze is much harder to remove than the traditional homogeneous haze. The dehazing performance tested on non-homogeneous haze can show the model’s capability well. We listed the dehazing quantitative performance of dehazing methods in Table 3. As shown in Table 3, DCP, BCCR, and NLD achieve a low quantitative dehazing performance. We note that DehazeNet achieves lower quantitative dehazing performance than DCP, BCCR, and NLD. learning-based methods^10,12,13,15,21^ achieve higher dehazing performance. Dehamer achieves the second-best quantitative dehazing performance. The proposed method demonstrates the best PSNR and SSIM among the listed dehazing methods. The results demonstrate the effectiveness of the proposed method, which benefits from the combination of CNN and transformer. We also show visual dehazed results of the proposed method and other state-of-the-art methods. As shown in Fig. 4, we can see that the traditional dehazing methods often over-enhance the dehazed results, which contain obvious color distortion. The learning-based methods tend to retain haze in dehazed results. In contrast to these methods, the proposed method often obtains visually pleasing dehazed results, which are vivid color and contain rich image details.

**Figure 6:**
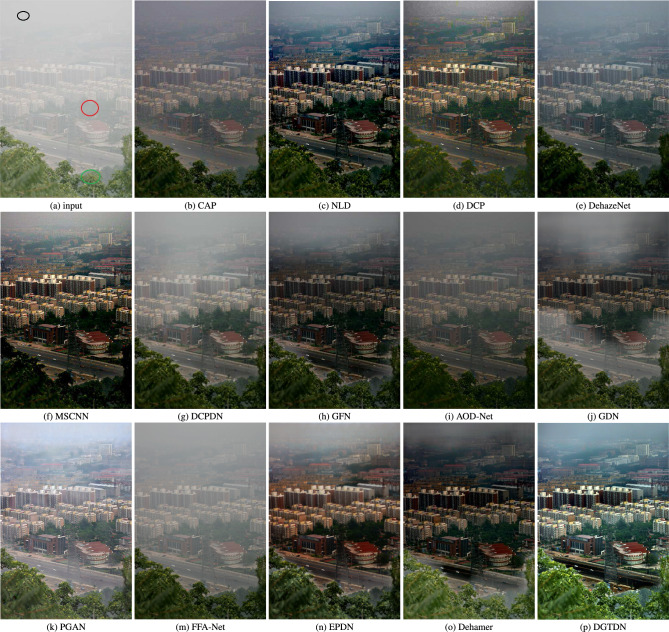
Visual results of dehazing methods. The dehazed result obtained by other state-of-the-art methods tends to show a hazed or dark appearance. The dehazed results of MSCNN and AOD-Net lose some details. In contrast, the proposed method often shows a sharp dehazed result and removes haze more completely.

**Figure 7:**
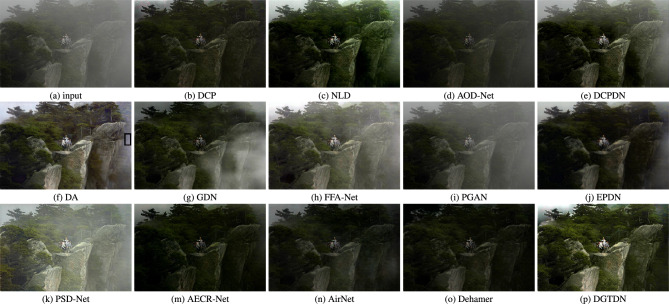
Visual results of some recently dehazing methods and the proposed method. The dehazed results obtained by other state-of-the-art methods tend to show a dark or hazed appearance. DA is designed for natural image dehazing with domain adaption. However, we note that the area marked with a black rectangle retains a lot of haze. In contrast, the proposed method often shows a colorful and sharp dehazed result and removes haze more completely.

**Table 4:** Density values for a natural hazy image in Fig. 7.

Input	DCP	NLD	FFA-Net	AECR-Net	AirNet	EPDN	DA	MSBDN	PSD-Net	Dehamer	DCPDN	PGAN	AOD-Net	Our
1.832	0.398	0.348	0.737	0.294	0.634	0.293	0.405	0.802	0.230	0.327	0.652	0.319	0.732	**0.204**

The best result is marked with bold.

**Table 5:** The quantitative results with different modules on the synthetic hazy dataset.

Metric	Model1	Model2	Model3	DGTDN
PSNR	25.83	36.49	36.88	36.68
SSIM	0.92	0.98	0.99	0.99

**Figure 8:**
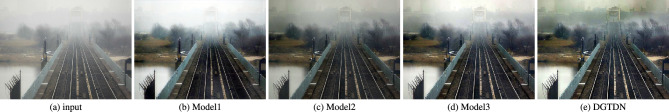
Visual results of different model configurations and the proposed method. The dehazed result obtained by other different models often retains haze or color distortion. The proposed method can remove haze more completely and obtain a more natural dehazing result.

**Table 6:** The quantitative results with different loss functions on the synthetic hazy dataset.

Metric	w/o $\mathscr {L}_{\text {perc}}$	w/o $\mathscr {L}_{\text {baseloss}}$	w/o $\mathscr {L}_{\text {con}}$	DGTDN
PSNR	34.29	34.79	24.06	36.68
SSIM	0.98	0.98	0.78	0.99

**Figure 9:**
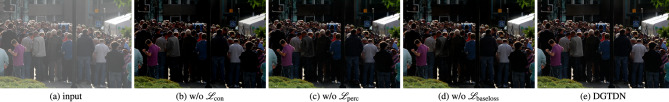
Visual results of models trained with different loss functions. The dehazed result obtained by other different models often retains haze or color distortion. The proposed method can remove haze more completely and obtain a more natural dehazing result.

### Experimental results on real-world haze images

To further show the performance, we choose some typical real-world hazy images. The density and distribution of haze in real hazy images are more multiplicative than in synthetic images. Hence, the real-world hazy image dehazing is a more challenging problem. In this part, we choose three hazy images, which include dense haze, large haze distribution, and dark haze images. These haze images can show the generalization and dehazing performance of deep-learning-based models.

Firstly, we conduct an experiment on a dense haze image. The dehazed results of state-of-the-art methods and the proposed method are shown in Fig. 5. As shown, we can see that the image tends to show dense haze over the whole image, which is hard for CNN-based dehazing methods. The dehazed results of AOD-Net^13^ and DCPDN^15^ tend to retain haze. The dehazed result of GFN^12^ contains visible color distortion and haze. The dehazed result of cGAN^49^ contains less color distortion than GFN and can remove haze better than AOD-Net, DCPDN, and GFN. We note that the dehazed results of EPDN, Dehamer, and the proposed method are better than other learning-based methods. We note that the area in the lake is not well dehazed in a result of EPDN. The proposed method can remove haze more completely than EPDN and Dehamer. Due to the fact the transformer can capture long dependency, which can boost the dehazing quality. The proposed method and Dehamer remove haze from dense haze images. The proposed method employs CNN to restore the image details, which makes the proposed method can restore more image details than Dehamer.

Secondly, we conduct an experiment on a hazy image with large haze distribution. This hazy image is a typical image, which has been employed to evaluate the dehazing performance widely. This image contains dense haze areas, a middle haze area, and light haze areas, which are marked using black, red, and green circles, respectively. Due to its large haze distribution, the learning-based methods often fail to remove haze well. As shown in Fig. 6, we note that the traditional methods^1,4^ often show a better dehazed results than learning-based methods^12,13,15^. The dehazed result of Non-local dehazing tends to lose image details and shows a dark appearance. The dehazed result of DCP^1^ tends to retain a small amount of haze. The dehazed results of CAP, DCPDN, FFA-Net, and AOD-Net tend to retain a large amount of haze. The dehazed results of GDN^20^ and GFN^12^ contain color distortion. Dehazenet and MSCNN are based on deep learning and Koschmieder’s law. We note that the dehazed result of MSCNN is better than DehazeNet, which can remove more haze. We also note that the dehazed result of MSCNN losses some image details. The dehazed result of PGAN^34^. However, we note the dehazed result of PGAN still contains haze. The dehazed results of EPDN and Dehamer can remove haze better. However, these methods tend to generate a dark dehazed result and tend to show some haze around the green circle area. The proposed method can remove haze more completely and keep the image details well.

Thirdly, we conduct an experiment on a more challenging image, which looks dark. The dehazed results of this image often have the problem of losing image details and retaining haze. As shown in Fig. 7, we can see that the dehazed result of DCP, AOD-Net, AECR, AirNet^14^, EPDN, and Dehamer tend to show a dark appearance. The dehazed result of DCPDN, FFA, and PGAN looks brighter. However, the dehazed results of these methods tend to retain haze in the dehazed result. The dehazed result of DA, PSD, and DGTDN can generate a much brighter dehazing result. However, the dehazed result of PSD tend to retain haze in the whole image while the result of DA tends to leave haze in a black rectangle and show a blur dehazed result. AirNet is based on the assumption that the whole image shares similar degradation. In contrast, the proposed method can remove haze more completely and obtain a sharp dehazed result. To show the quality of dehazed results obtained by the proposed method and other dehazing methods quantitatively, we use the metric proposed in^50^. As shown in Table 4, we can see that the proposed method can remove haze better than other dehazing methods.

### Ablation studies

**Table 7:** Running states of the state-of-the-art dehazing methods and proposed methods on the 500 images with 256$\times$256 size. The running states include language, platform, execution time, parameters, consumption of GPU memory.

Method	DCP	NLD	MSCNN	DehazeNet	GFN	DCPDN	EPDN	DA	FFA-Net
Language	Matlab					Python			
Platform			Caffe			PyTorch			
Time (s)	0.302	3.416	0.102	**0.051**	2.740	0.106	0.156	0.096	2.431
Parameters	–	–	**8.01 × ** $10^3$	8.02×$10^3$	5.14 × $10^5$	6.69 × $10^7$	1.74 × $10^7$	5.46 × $10^7$	4.46 × $10^6$
Memory (G)	–	–	**0.019**	1.282	0.169	1.65	1.73	0.939	1.107

Method	MSBDN	AirNet	PSD	AECR	PGAN	GDN	Dehamer	Model3	DGTDN
Language	Python								
Platform	PyTorch								
Time (s)	0.462	0.772	0.132	0.053	0.268	0.219	0.298	0.169	0.061
Parameters	3.14 × $10^7$	7.61 × $10^6$	3.31 × $10^7$	2.61 × $10^6$	1.14 × $10^7$	9.58 × $10^5$	1.32 × $10^8$	4.64 × $10^6$	4.65 × $10^6$
Memory (G)	0.909	1.025	0.855	0.825	2.345	1.138	1.862	0.868	0.726

The best result is marked with bold.

To the effectiveness of the proposed module in DGTDN, we design a series of experiments. Firstly, we design a model to show the effectiveness of the transformer. We remove the transformer from the proposed model, and keep other parts unchanged, we term it as model1. Secondly, we show the effectiveness of the DetailNet. We remove the DetailNet from the proposed model, and keep other parts unchanged, we term it as model2. Finally, we show the effectiveness of the GuidedFilterNet, which can boost the dehazing speed of the proposed model. To show the influence of the GuidedFilterNet, we design a model which removes the GuidedFilterNet and keeps other parts unchanged, we term it as model3. We show the quantitative comparison in Table 5 and a visual example in Fig. 8. As shown in Table 5, we can see that the model1 achieves the lowest dehazing performance due to the limitation of the receptive field. As we can see that the BaseNet can boost the dehazing performance dramatically, which shows the transformer module is necessary for dehazing. The transformer module can improve the dehazing performance by enlarging the receptive field. We note that the application of guided filter reduces the dehazing performance. However, it is necessary to improve the dehazing speed while only reducing the dehazing performance slightly. We show the difference dehazed result of model1, model2, model3, and the proposed model in Fig 8. We can see that model1 cannot remove haze in remote areas, which are dense haze. The transformer module is necessary for removing dense haze areas. By adding the DetaiNet, we can see that the model can remove haze more completely. The guided filter improve the dehazing quality in remote areas.

To show the influence of loss functions, we design an ablation that involves the models are trained with different losses. First, we train the model without $\mathscr {L}_{\text {perc}}$. Second, we train the proposed model without $\mathscr {L}_{\text {baseloss}}$. Third, we train the proposed model without $\mathscr {L}_{\text {con}}$. We show the quantitative results in Table 6. As shown, $\mathscr {L}_{\text {con}}$ is critical to obtain a high quantitative dehazing result. $\mathscr {L}_{\text {con}}$ is designed to boost the details of the dehazed results. $\mathscr {L}_{\text {con}}$ is designed to make the dehazed results similar to the ground truths. $\mathscr {L}_{\text {baseloss}}$ is used to reduce the difficulty of dehazing problem, which can boost the dehazing quality. We also show dehazed results of the model trained with different loss functions in Fig. 9. As shown, we note that the model trained without $\mathscr {L}_{\text {con}}$ obtains a dehazed result that losses image details. The dehazed results obtained by models trained without $\mathscr {L}_{\text {baseloss}}$ or $\mathscr {L}_{\text {perc}}$ generate results with color distortion or over-enhancement. As shown in Fig. 9, the model trained with all losses can generate high quality dehazing results.

### Analysis of run states

We test the dehazing speed of dehazing methods on 500 images with size $256\times 256$. The test hazy images are from the outdoor part of RESIDE, we resize these images into a fixed size ($256\times 256$). We conduct the experiment on a notebook, which is equipped with an Intel(R) Core i5 CPU@2.3GH, 8GB memory, and a 3GB RTX 1060 GPU. The average running times of state-of-the-art dehazing methods and the proposed method is shown in Table 7. The traditional dehazing methods^1,4^ are slower than learning-based methods. These methods are executed without parallelization technology, which increases the execution time. The early learning-based method^11^ is faster. However, the dehazing performance of this method is poor. The proposed method achieves state-of-the-art dehazing performance while keeping a lower execution time. In addition, we show the rum states of each method in Table 7. The running states include language, platform, execution time, parameters, and consumption of GPU memory.

As shown in Table 7, the proposed model has a suitable parameter number and consumes suitable GPU memory, while achieving the highest quantitative performance. We also show the effectiveness of the GuidedFilterNet, which can reduce the execution time and GPU memory compared with model3. As shown in Table 5, the proposed method is with almost no visible degradation compared with model3. We can obtain the conclusion the GuidedFilterNet can improve the execution speed while avoiding performance degradation.

### Extended applications

Based on the fact the proposed model can capture the local and global features jointly, we can apply the proposed model to solve the problem, such as underwater enhancement^51–55^, detain^56^, and human image generation^57^. Single image underwater enhancement is a challenging problem due to its ill-posed nature. The global information and local details of underwater images are degraded by water, which results in the degeneration of each pixel may be different. Based on this observation, the high-performance model requires global features to capture the degeneration. The underwater enhancement also needs to restore the fine details, which requires the local features. The underwater enhancement is similar to dehazing, which also needs global and local features jointly and a low compute resource requirement. The proposed model can capture the global and local features jointly, which also can be applied to underwater enhancement.

## Conclusion

Deep Guided Transformer Dehazing Network (DGTDN) is proposed based on the transformer and guided filter, which boosts the speed of transformer-based dehazing methods and the image quality of dehazed result. The proposed model consists of BaseNet, DetailNet, and GuidedFilerNet. BaseNet and DetailNet are proposed to capture the local and global features jointly. To boost the advantages of the transformer module and the CNN module, we employ the transformer module to predict the base layer of a clean image, and the CNN module to predict the detail layer. To address the dehazing speed problem of the transformer module, we employ the guided filter model to perform a joint up-sampling, which can improve the dehazing speed while keeping the quality of dehazed result. We show the effectiveness of the proposed method by comparing it with state-of-the-art dehazing methods on real and simulated haze images. We also show the effectiveness of the novel modules by comparing the performance of the different architectures and loss functions. In the future, we will study strategies of combining CNN and the transformer, which is critical to capture the local and global features. We also will study the domain shift of simulated-haze and real-haze images, which is critical to boost the dehazing performance on real haze images.

## Data Availability

The datasets generated and/or analyzed during the current study are available in the RESIDE repository, which can be found at: https://sites.google.com/view/reside-dehaze-datasets/reside-standard. The natural hazy images are from: http://live.ece.utexas.edu/research/fog/fade_defade.html and https://www.cs.huji.ac.il/w~raananf/projects/dehaze_cl/results/.
